# 1853. Examining the Role of Race-Ethnicity and Neighborhood Socioeconomic Disadvantage in Influenza Vaccine Uptake

**DOI:** 10.1093/ofid/ofad500.1681

**Published:** 2023-11-27

**Authors:** Anuja Sarode, Caitlin Speyer, Daniel J Tisch, Elie Saade, Robert Salata

**Affiliations:** University Hospitals, Brecksville, Ohio; University Hospitals, Brecksville, Ohio; Case Western Reserve University, Cleveland, Ohio; Case Western Reserve University, Cleveland, Ohio; Case Western Reserve University Hospitals, Cleveland, OH

## Abstract

**Background:**

Negative influenza-associated outcomes disproportionately impact non-White individuals. Targeted influenza vaccine coverage could improve outcomes in minority groups. This study examined the role of race-ethnicity and resource inequity in vaccine coverage.

**Methods:**

We analyzed data from all outpatient visits at University Hospitals CMC from October 01, 2022 to February 28, 2023. Race, ethnicity, age, sex, income, home address, ICD diagnosis, and influenza immunization records were collected. Patients who received an influenza vaccine between July 01, 2022 and February 28, 2023 were considered immunized. A 9-digit patient zip code was used to obtain state Area Depravity Index (ADI) data provided by University of Wisconsin School of Medicine and Public Health. The state ADI ranges from 1 to 10, with group 1 being the least disadvantaged and 10 being the most disadvantaged. We used logistic regression analysis to estimate the odds for being vaccinated predicted by race-ethnicity, state ADI, age, and sex.

**Results:**

Out of 301,572 hospitalizations, 59.0% were women, 81.2% were White non-Hispanic, 16.0% were Black non-Hispanic, and 2.7% were Hispanic. Black non-Hispanics were most disadvantaged with a mean State ADI of 7.54, followed by Hispanics at 5.64. White non-Hispanics were least disadvantaged with a mean State ADI of 3.99 (p< 0.001)(Table 1). Black non-Hispanics were disproportionately represented in the most disadvantaged areas (27.93%). Black non-Hispanics were 38% less likely to be vaccinated than White non-Hispanics. After ADI, sex, and age adjustment, Black non-Hispanics were 11% less likely to be vaccinated (OR: 0.89; 95% CI: 0.868 - 0.912). With each point increase in State ADI, vaccination rate decreased by 7% (OR: 0.93; 95% CI: 0.928 – 0.933)(Table 3). Regression analysis suggested significant interaction between race-ethnicity and State ADI.

**Figure:**
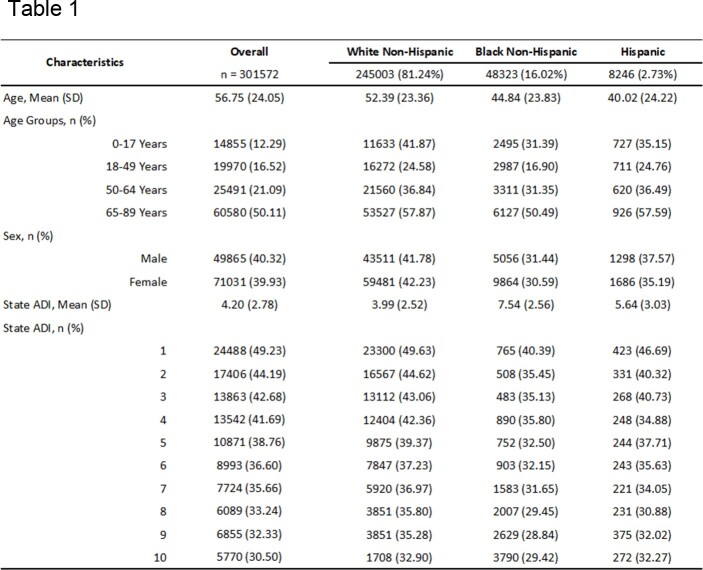

Influenza Vaccination by individual characteristics within overall population and race-ethnicity

**Figure:**
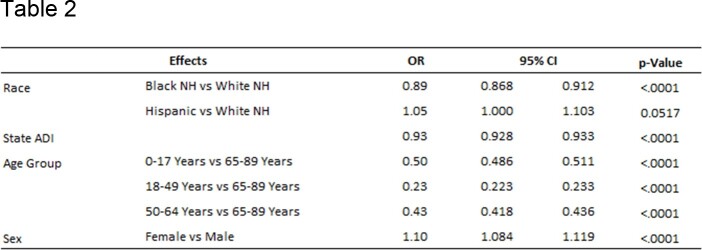

Influenza vaccination by race-ethnicity adjusting for State ADI, age, and sex

**Figure:**
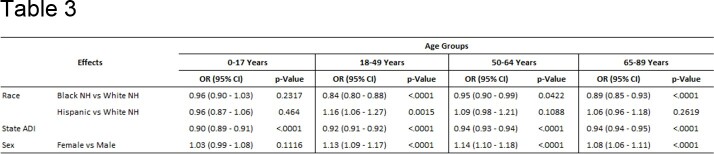

Influenza vaccination by race-ethnicity adjusting for State ADI and sex, and stratified by age groups

**Conclusion:**

This cross-sectional study found racial and ethnic disparities in rates of influenza vaccination. The effect of race-ethnicity on vaccination rate is dependent on ADI. These data identified subgroups on which to focus future influenza prevention efforts. The generalization of the study is limited and similar analysis from other areas and vaccines would be beneficial.

**Disclosures:**

**Elie Saade, MD, MPH, FIDSA**, Envision Pharma: Speaker, Presenter|Johnson and Johnson: Speaker, Travel, Lodging|Protein Sciences Corp: Grant/Research Support|Sanofi Pasteur: Speaker, Travel and Lodging

